# Electrophysiological and Morphological Characterization of Contact Chemosensilla in Adults and Larvae of the Butterfly, *Atrophaneura alcinous*

**DOI:** 10.3390/insects13090802

**Published:** 2022-09-02

**Authors:** Kazuko Tsuchihara, Takuma Takanashi, Kiyoshi Asaoka

**Affiliations:** 1 Department of Information Science, Faculty of Liberal Arts, Tohoku Gakuin University, Sendai 981-3193, Japan; 2 Tohoku Research Center, Forestry and Forest Products Research Institute, Morioka 020-0123, Japan; 3 Institute of Agrobiological Sciences, National Agriculture and Food Research Organization, Tsukuba 305-8634, Japan

**Keywords:** chemoreception, taste, butterfly, sensilla

## Abstract

**Simple Summary:**

In butterflies, oviposition and feeding are induced by the recognition of host plant compounds with contact sensory organs called chemosensilla. Oviposition behavior of the butterfly *Atrophaneura alcinous* involves detection of two plant compounds—an alkaloid and a monosaccharide—using tarsal contact chemosensilla of the foreleg. Feeding behavior of the adults and larvae involves detection of sucrose, sodium chloride, and other chemicals. We examined the distribution and electrophysiological response of contact chemosensilla in *A. alcinous* adults and larvae. In adults, tarsal contact chemosensilla were classified into long- and short-type sensilla. Long-type sensilla were distributed widely much more in females than in males, whereas short-type sensilla were found at the edge of the tarsi in a similar manner in both sexes. Long- and short-type sensilla showed different responses to methanol extracts of host plant compounds. Our findings suggest that the adult butterfly discriminates between host plant compounds using long-type sensilla during oviposition and recognizes the taste using short-type sensilla. In the larval mouthparts, contact chemosensilla were present on the maxillary galea and epipharynx. Electrophysiological responses of chemosensilla suggest that larvae can discriminate between host plant compounds.

**Abstract:**

Distribution and electrophysiological responses of contact chemosensilla were examined in the Aristolochiaceae-feeding butterfly *Atrophaneura* *alcinous*. In adult butterflies, tarsal contact chemosensilla of the foreleg were classified into two groups based on length: long- and short-type sensilla. Long-type sensilla were distributed much more widely in females than in males, whereas short-type sensilla were found at the edge of the tarsi in a similar manner in both sexes. Taste responses of the long- and short-type sensilla to methanol extracts of *Aristolochia debilis* and *Citrus* spp. were recorded. *Aristolochia* debilis extracts evoked spikes with different amplitudes, whereas Citrus spp. extracts evoked spikes with a single amplitude in the long-type sensilla. Short-type sensilla did not respond to either extract. Moreover, we recorded responses to different concentrations of sucrose and NaCl. Results suggest that adult butterflies can discriminate the taste of host plant components from other chemicals using long-type sensilla during oviposition and may recognize diets containing sugar and salts during feeding using short-type sensilla. In the larval mouthparts, there were lateral and medial styloconic sensilla on the maxillary galea and epipharyngeal sensillum on the epipharynx. Electrophysiological responses of these sensilla suggest that larvae can discriminate between host plant compounds.

## 1. Introduction

The chemosensory organs of insects recognize various plant compounds that stimulate different behaviors [1,2,3,4,5,6,7,8]. Butterflies respond to environmental chemicals to feed and lay eggs [9]. Swallowtail butterflies are particularly selective when laying eggs on larval host plants; for example, *Papilio xuthus* targets *Citrus* spp., whereas *Atrophaneura alcinous* targets *Aristolochia debilis*. In butterflies, oviposition on host plants is evoked through species-specific combinations of chemosensory systems and plant compounds [7,10,11,12]. Female butterflies have a toothbrush-like dense cluster of sensilla on the ventral surface of the fifth tarsal segment of the foreleg and carry more sensilla than males [13,14].

Studies on *A. alcinous* have identified two major oviposition stimulants: the alkaloid aristolochic acid and the monosaccharide sequoyitol [6]. Tsuchihara et al. [15] identified a chemoreceptive protein that binds to the ligand responsible for stimulating oviposition in butterflies for the first time. In females of *A. alcinous*, the chemoreceptive protein in the contact chemosensilla recognizes aristolochic acid [16,17,18]. Moreover, electrophysiological responses to oviposition stimulants have been recorded from the tarsal contact chemosensilla of the forelegs in *A. alcinous* and *P. xuthus* [15,17,18,19]. In *A. alcinous*, different spike amplitudes were observed when sensilla were in contact with aristolochic acid. When sensilla were stimulated with sequoyitol, spikes with a single amplitude were observed [18]. When sensilla were stimulated with a mixture of aristolochic acid and sequoyitol, two different spikes were observed. However, it is difficult to discriminate between the two spikes because of their low sensitivities to these compounds [18]. In this study, we recorded the electrophysiological responses of *A. alcinous* to host plant extracts, including aristolochic acid and sequoyitol.

In contrast, in lepidopteran larvae, contact chemoreceptors are located on the mouthparts and are critical for food-selection behavior. The electrophysiological characteristics of contact chemoreceptors have been investigated in several species [20]. Recently, chemosensory receptors have been identified in the larvae of the swallowtail butterfly *Papilio hospiton* [21].

Chemosensory studies in the adults of swallowtail butterflies have been investigated in *A. alcinous* and *Papilio* species [9,10,11,12,15,16,17,18,19,20,21]. However, electrophysiological studies on *Papilio* species are limited [15,17,18,19,20,21]. For this study, the chemosensory system of *A. alcinous* was investigated to understand host plant selection in swallowtail butterflies. Accordingly, the morphological and electrophysiological characteristics of contact chemosensilla were investigated in *A. alcinous* adults and larvae. Our findings revealed some sex differences in sensilla characteristics on the fifth tarsomere in adults. Considering these findings, electrophysiological responses to plant extracts and selective plant compounds were recorded and analyzed, and the role of contact chemosensilla for oviposition and feeding are discussed.

## 2. Materials and Methods

### 2.1. Insects

Adult butterflies of *A. alcinous* were collected from the field in Tsukuba, Ibaraki, Japan. The collected butterflies were housed in transparent plastic boxes with *A. debilis* leaves. All eggs laid on *A. debilis* leaves were collected and stored at 20 °C. After hatching was complete, the larvae were fed *A. debilis* leaves until pupation. During this time, they were maintained at 25 °C under 16 h/8 h light/dark conditions. The pupae were maintained under these conditions until adult emergence. Adult butterflies were reared at 25 °C and fed with a 15% sucrose solution for 3 days. Two-day-old female butterflies were mated by hand-pairing females with male butterfly individuals.

For morphological (male: *n* = 8; female: *n* = 16) and electrophysiological (male: *n* = 8; female: *n* = 16) experiments, unmated male and mated female adult butterflies were selected 3–7 days after emergence. For larval morphological (*n* = 8) and electrophysiological (*n* = 16) experiments, the fifth instar stage was used either 1 or 2 days following molting.

### 2.2. Stimulants

Adult female butterflies were predicted to discriminate between host plant and nonhost plant compounds. Therefore, we examined the electrophysiological responses to methanolic extracts of the host plant *A. debilis* and the nonhost plant *Citrus* spp. Whole leaf extracts were prepared as described by Nishida [6]. The leaves and stems of *A. debilis* and the leaves of *Citrus* spp. were extracted using 100% methanol. The extraction continued for 1 month. Methanol was evaporated and the residues were dissolved in 10% ethanol with equivalent water content to that of fresh leaves. This extract was known as “1.0 gle.”

Sodium chloride (NaCl), sucrose, and ethanol were purchased from WAKO Pure Chemical Industries. Aristolochic acid (Sigma-Aldrich, St. Louis, MO, USA) and sequoyitol (MedChemExpress, Princeton, NJ, USA) were used as stimulants to record the electrophysiological responses in larvae. All stimulants—with the exception of 200 mM NaCl—were dissolved in 20 mM NaCl. The solutions were then used to obtain electrophysiological recordings for conductivity. All stimulants contained 10% ethanol, which is an optimal concentration to dissolve leaf extract residues, minimizing the negative effect of ethanol on the response in electrophysiological recordings.

We selected different concentrations of sucrose because adults and larvae have different sensitivities to sucrose. The optimal concentration was 40 mM sucrose in adults and 100 mM sucrose in our preliminary experiments. We used 15 mM sequoyitol and 1 mM aristolochic acid in larvae based on previous study results [18].

### 2.3. Preparation for Electron Microscopy

To prepare samples for scanning electron microscopy, the tarsi were cut from adult forelegs using scissors. Maxilla and labrum were cut from fifth instar larvae. The samples were air dried, coated with gold, and observed under the JSM-6301F scanning electron microscope (SEM) (JEOL, Tokyo, Japan).

### 2.4. Electrophysiological Experiments

Before obtaining the recordings from tarsal contact chemosensilla of adult butterflies, the butterflies were anesthetized at 4 °C for approximately 1 h. Then, they were fixed between two plastic plates, ventral side up, and secured using cellophane film and dental wax. A pair of forelegs was adhered on a double-sided tape, with fixed ventral side up. Next, the tarsus was immobilized using a vinyl tape cut into thin strips.

The tip-recording method [22] was used to record action potential responses from each contact chemosensillum. A glass capillary with a tip diameter of 5–10 µm, created using a P-97 puller (Sutter Instrument Co., Novato, CA, USA), was filled with the stimulant solution and used for stimulating and recording electrodes simultaneously. The stimulation period was 2 s. To avoid stimulus adaptation, a stimulation-free interval of at least 5 min was provided between each stimulus. In addition, tips of stimulated sensilla were washed using a glass electrode filled with distilled water.

A platinum wire inserted into the capillary solution was then connected to the TastePROBE amplifier (Syntech, Hilversum, The Netherlands). Electric signal responses were recorded on a computer through an IDAC-2 converter (Syntech) and analyzed using the AutoSpike32 software (Syntech). A stainless-steel needle with a sharply-polished tip was inserted into the proximal part of the tarsus and used as an indifferent electrode.

To record contact chemosensilla of the larval mouthparts, a living larva was mounted on a silicone tube with an inner diameter of 9 mm and a vertical slit. Only the head protruded from the tube, with the maxilla or epipharynx (i.e., the ventral part of the labrum) immobilized with a plastic paraffin film cut into thin strips. Electrophysiological recording was performed in the same manner as described for adult tarsi, with the exception that the indifferent electrode was inserted into the proximal part of the maxilla or epipharynx.

## 3. Results

### 3.1. Characterization of Sensilla Types in the Forelegs of Male and Female Adults

**Figure 1:**
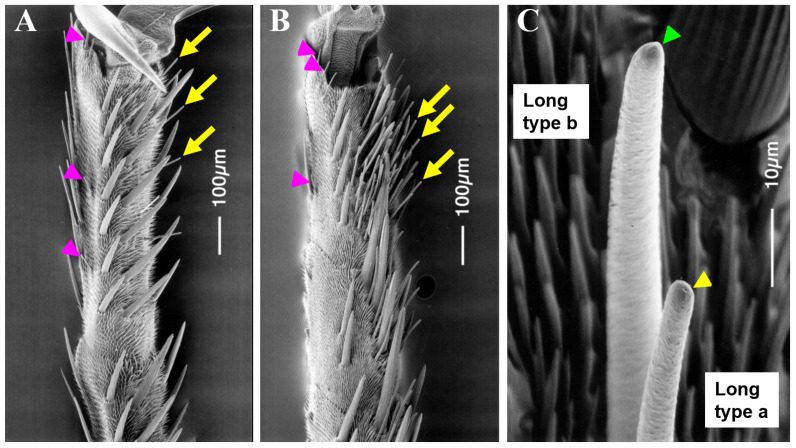
Scanning electron micrographs of contact chemosensilla of the fifth tarsomeres in the forelegs of adult *Atrophaneura alcinous*. (**A**) Ventral side of the fifth tarsomere in the male. (**B**) Ventral side of the fifth tarsomere in the female. Arrows and arrowheads indicate long- and short-type sensilla, respectively, in (**A**,**B**). (**C**) Morphological differences between long-type a sensilla and long-type b sensilla of the fifth tarsomere in the female. Long-type sensilla with a single pore at the tip (yellow arrowhead) and long-type b sensillum with a small protuberance at the tip (green arrowhead).

Contact chemosensilla in female and male forelegs of *A. alcinous* individuals were classified into two morphological types based on their length: long type (hereafter abbreviated as “L”: 50–100 µm) and short type (“S”: 30–50 μm) (Figure 1A,B). L sensilla were distributed widely on the ventral surface of the first to fifth tarsomeres. Notably, female tarsi have more sensilla in the fifth tarsomere than males. The number of L sensilla was greater in females (~75 sensilla; *n* = 16) than in males (~7 sensilla; *n* = 16) (Figure 1). In contrast, S sensilla were found at the side of each tarsomere, with 2–10 in each tarsomere (*n* = 16). No difference between sexes was observed in the number of S sensilla on the tarsomere. It was noted that each sensillum, for both types, formed a small pore at the tip (Figure 1C). The thicknesses and lengths of the sensilla were similar.

**Figure 2:**
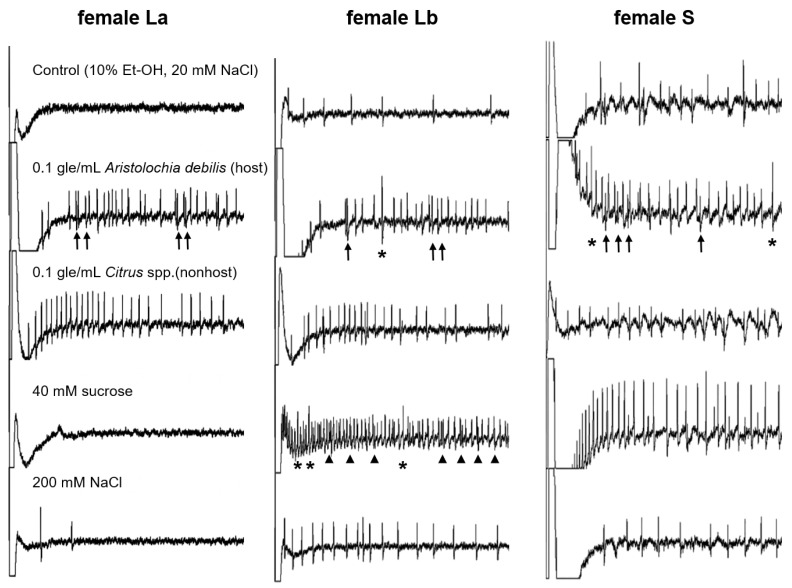
Electrophysiological responses to stimulants from the three types of female contact chemosensilla. Individual responses covered 0.5 s after stimulus onset. Stimulation with the extract of the host plant *Aristolochia debilis* caused one cell to fire at a constant rate and another (indicated by arrows) to fire irregularly and less frequently in all sensilla. Stimulation with sucrose caused one cell to fire at a constant rate and another (indicated by arrowheads) to fire irregularly and less frequently in long-type b sensilla. Asterisks indicate doublets of two spikes from two different cells. La: Long-type a sensilla; Lb: Long-type b sensilla; S: short-type sensilla. “gle/mL” indicates gram leaf equivalent in 1 mL of control solution.

**Figure 3:**
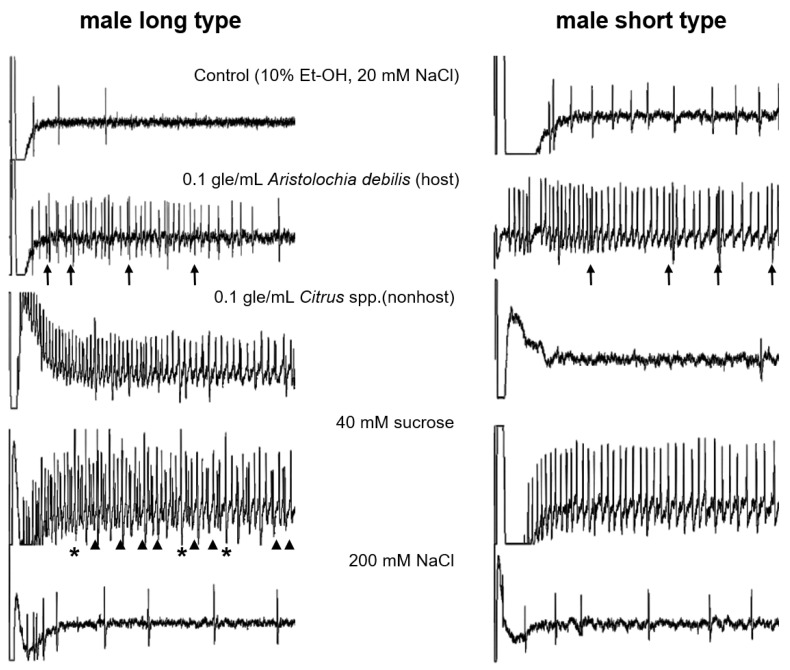
Electrophysiological responses to stimulants from male sensilla. Individual responses covered 0.5 s after stimulus onset. Stimulation with extracts of host plants *Aristolochia debilis* caused one cell to fire at a constant rate and another (indicated by arrows) to fire irregularly and less frequently in the long-and short-type sensilla. Stimulation with sucrose caused one taste cell to fire at a constant rate and another (indicated by arrowheads) to fire irregularly and less frequently in the long-type sensilla. Asterisks indicate doublets of two spikes from two different cells. “gle/mL” indicates gram leaf equivalent in 1 mL of control solution.

According to the electrophysiological responses to sucrose, L sensilla of the female tarsus were classified into two subtypes (Figure 2). The stimulation of the L sensillum in a female with 40 mM sucrose evoked a spike train in 5 of 75 sensilla (7.0%). Most of these 75 sensilla (93.0%) showed no response to stimulation with sucrose. In this study, the majority of female L sensilla that did not respond to sucrose stimulation were designated “La”; whereas the remainder, which showed a clear response to 40 mM sucrose, were designated as “Lb” SEM analysis revealed the presence of a small protuberance on the tip of the Lb sensillum (Figure 1C). Conversely, in the male tarsus, a single type of L sensillum clearly responded to 40 mM sucrose, which is similar to the response observed in Lb sensilla in females (Figure 2 and Figure 3).

### 3.2. Electrophysiological Response to Stimuli in Adults

Electrophysiological responses were recorded from the sensilla of male and female tarsi. Preliminary recordings revealed that sensilla in the proximal parts of the fifth tarsomere exhibited high sensitivities to the methanolic extracts of *A. debilis* (hereafter referred to as “host plant extract”) (*n* = 2).

Representative responses recorded from the three types of female sensilla—La, Lb, and S—stimulated with extracts and compounds are illustrated in Figure 2. Stimulation of La sensilla with the host plant extract evoked a train with two different spikes, causing one sensory cell to fire at a constant rate and another to fire irregularly and less frequently. Electrophysiological recordings revealed spikes with a single amplitude from La sensilla in response to methanolic extracts of the nonhost plant *Citrus* spp. (hereafter referred to as “nonhost plant extract”). Stimulation of La sensilla did not evoke responses from either 40 mM sucrose or 200 mM NaCl. Furthermore, no responses were recorded in La sensilla to stimulation with the control solution containing 10% ethanol and 20 mM NaCl.

Stimulation of Lb and S sensilla with the host plant extract evoked two spikes with different firing rates (Figure 2). Stimulation of female Lb and S sensilla with the nonhost plant extract evoked spikes with a single amplitude. Moreover, stimulation of Lb and S sensilla with 40 mM sucrose or 200 mM NaCl evoked spikes with a single amplitude. The control solution, containing 10% ethanol and 20 mM NaCl, evoked only a weak response in Lb and S sensilla.

Representative responses recorded from the two types of male sensilla—L and S—with the stimulants used for females are shown in Figure 3. Stimulation of L sensilla with the host plant extract evoked spikes with two different amplitudes. Electrophysiological recordings revealed single spike amplitudes from L sensilla in response to the nonhost plant extract. Stimulation of L sensilla with 40 mM sucrose or 200 mM NaCl evoked a notable response (Figure 3). In particular, 40 mM sucrose evoked a strong response, with two different spike amplitudes.

**Figure 4:**
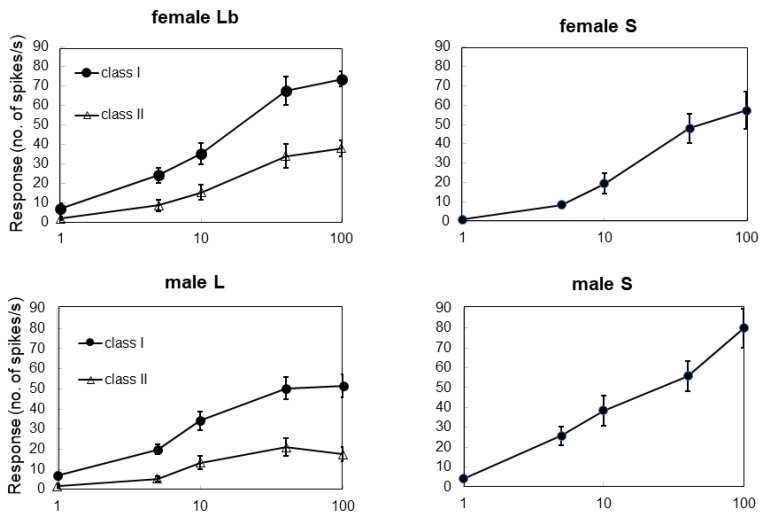
Dose dependency of response to sucrose in male and female sensilla. The responses were observed in both cells (class I and II) in female long-type sensilla b (female Lb) and male long-type sensilla (male L) and for one cell in female short-type sensilla (female S) and male short-type sensilla (male S) (*n* = 6–21).

Responses to different doses of sucrose were examined in Lb, L, female S, and male S sensilla (Figure 4). In both sexes, each cell with a single spike amplitude from S sensilla showed significant dose dependency (Spearman’s rank correlation: female S, ρ = 0.88, *p* < 0.01; male S, ρ = 0.85, *p* < 0.01). In both sexes, two different cells with two distinct spike amplitudes, namely class I and class II, from Lb and L sensilla showed significant dose dependency (Spearman’s rank correlation: class I of Lb, ρ = 0.89, *p* < 0.01; class II of Lb, ρ = 0.85, *p* < 0.01; class I of L, ρ = 0.72, *p* < 0.01; class II of L, ρ = 0.48, *p* < 0.01) (Figure 4).

### 3.3. Different Cells Respond to Stimulants of Host or Nonhost Plants

A sensory cell typically produces single spike amplitudes with a regular frequency. Therefore, the number of responding cells can be determined based on the amplitude and temporal pattern of the spikes. To determine whether the same sensory cells in La sensilla were responding to host and nonhost plant extracts, response patterns were analyzed.

**Figure 5:**
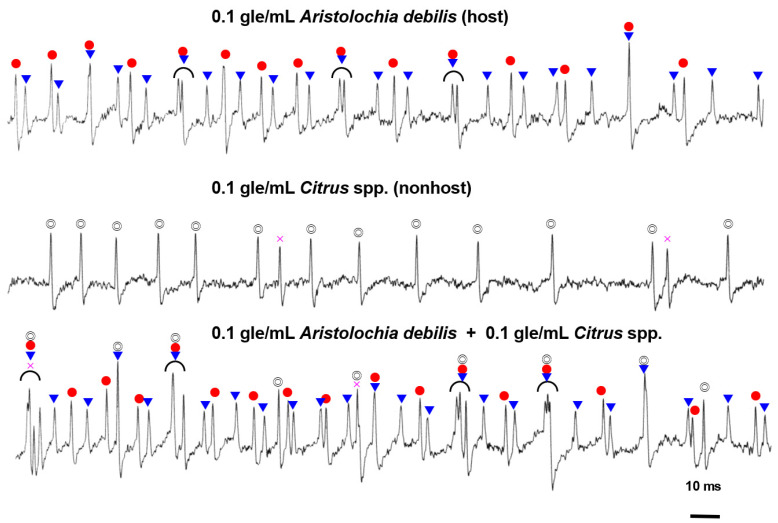
Electrophysiological responses to extracts of host and nonhost plants and their mixture from female long-type sensilla a with no recorded sensitivity to sucrose (La). Responses from three different classes of cells could be discriminated. ●: class 1; ▼: class 2; ◎: class 3; ×: unidentified. “gle/mL” indicates gram leaf equivalent in 1 mL of control solution.

In response to the host plant extract, two different cells responded with two different spike amplitudes, namely class 1 and class 2 (Figure 5). Stimulation of La sensilla with the nonhost plant extract evoked single spike amplitudes. To evaluate whether the same or different cells respond to different extracts, a mixture of host and nonhost plant extracts was used for stimulation. The results revealed that different cells were activated by the host and nonhost plant extracts because spike amplitudes and temporal patterns of three different cells were identified (Figure 5). We discriminated the spikes based on firing frequencies, amplitudes, and shape observed with the naked eye. Responses to different doses of methanolic extracts of the host plant were found in female and male long-type sensilla (data not shown).

### 3.4. Contact Chemosensilla Are Present on Larval Mouthparts

**Figure 6:**
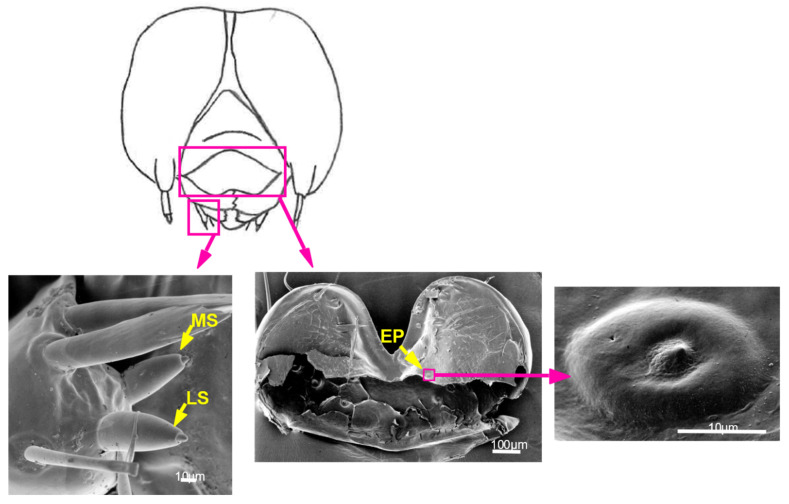
Scanning electron micrographs of contact chemosensilla on the mouthparts of the larvae of *Atrophaneura alcinous.* LS: lateral styloconic sensilla; MS: medial styloconic sensilla; EP: epipharyngeal sensilla.

Three types of contact chemosensilla were observed on the maxilla and epipharynx in the mouthparts of *A. alcinous* larvae. This is well known among lepidopteran larvae and was confirmed in this study using SEM. Two types of sensilla styloconica were observed on the maxillary galea: a lateral styloconic (LS) sensillum and a medial styloconic (MS) sensillum (Figure 6). In the epipharynx, a sensillum coeloconicum was observed, referred to as an epipharyngeal (EP) sensillum (Figure 6).

**Figure 7:**
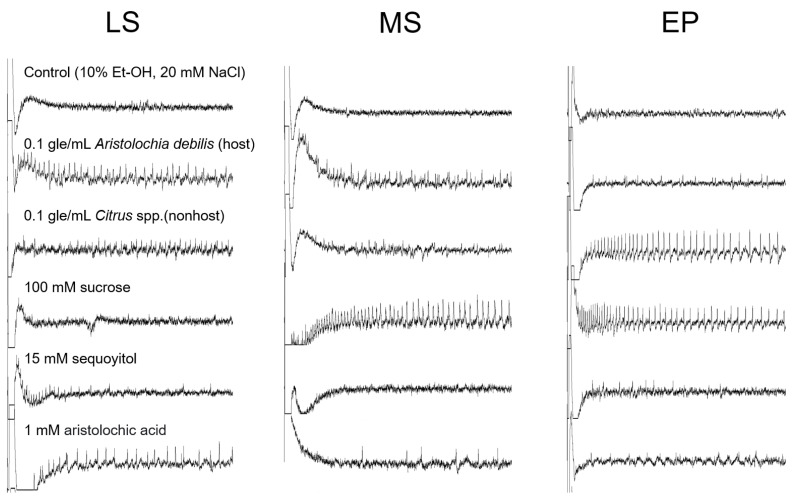
Electrophysiological responses to stimulants from larval sensilla. Individual responses covered 0.5 s after stimulus onset. LS: lateral styloconic sensilla; MS: medial styloconic sensilla; EP: epipharyngeal sensilla. “gle/mL” indicates gram leaf equivalent in 1 mL of control solution.

Electrophysiological responses were recorded from the three types of sensilla in 16 larvae. Representative responses from the three contact chemosensilla to host and nonhost plant extracts, 100 mM sucrose, 15 mM sequoyitol, 1 mM aristolochic acid, and control solution (10% ethanol and 20 mM NaCl) are shown in Figure 7. LS and MS sensilla differentially responded to sucrose and sequoyitol. Stimulation of LS sensilla with 100 mM sucrose evoked no spikes. However, MS sensilla evoked a train of spikes with a single amplitude. Conversely, stimulation of LS sensilla with 15 mM sequoyitol evoked spikes with a single amplitude, and no spikes were evoked in MS sensilla.

Stimulation of EP sensilla with the nonhost plant extract and 100 mM sucrose evoked a train of spikes with a single amplitude and regular temporary pattern. Few small spike amplitudes were observed in response to 15 mM sequoyitol. Stimulation of LS, MS, and EP sensilla with the host plant extract evoked spikes with multiple different amplitudes. Moreover, 1 mM aristolochic acid evoked spikes with a single amplitude in LS sensilla. Stimulation of LS and MS sensilla with the nonhost plant extract evoked one or few spikes with small amplitudes.

## 4. Discussion

Adult females of *A. alcinous* had three types of contact chemosensilla in foreleg tarsi, namely La, Lb, and S sensilla (Table 1). In La sensilla, two cells with different spike amplitudes (class 1 and class 2) responded to the host plant extract. In addition, one cell with class 3 spike in La sensilla responded to the nonhost plant extract. Lb sensilla appeared to contain one cell responding to the host plant extract and another responding to the nonhost plant extract. Furthermore, Lb sensilla contained two cells that responded to sucrose (Figure 4). Thus, one of these cells may be similar to the cell that responds to the host or nonhost plant extract.

In contrast, no cells in La sensilla responded to sucrose. This indicates that the three cells with three different classes of spike respond to a compound in the host or nonhost plant extract other than sucrose. Because La sensilla comprise the biggest number of tarsal sensilla, they can discriminate between the host and nonhost plants during oviposition. In female S sensilla, only one cell responded to sucrose. Adult males have two types of sensilla, L and S (Table 1). Male S sensilla only have one cell that responded to sucrose. Two cells responded to sucrose in male L sensilla and female Lb sensilla and showed similar characteristic response patterns to all stimulants. Because *A. alcinous* is a nectar-feeding butterfly, male L sensilla and female Lb sensilla—in addition to male and female S sensilla—function in host plant discrimination and feeding. Thus, *A. alcinous* can recognize specific plant compounds using different types of sensilla, depending on the oviposition or feeding state.

For oviposition, it has been reported that the females of *A. alcinous* detect two oviposition stimulants (aristolochic acid and sequoyitol) in the host plant *A. debilis* [6]. Although each compound alone elicits a low oviposition response, a combination of the two compounds synergistically induces oviposition. According to Tsuchihara [18], *A. alcinous* contains two cells that specifically respond to the two compounds in female long-type sensilla: one cell to aristolochic acid and another to sequoyitol. This observation is consistent with the findings of our study in which two cells in La sensilla responded to the host plant extract, including the two compounds (Table 1). Thus, we hypothesize that at least two cells responding to specific compounds induce oviposition on the host plant in *A. alcinous*. Furthermore, at least one cell in La sensilla participates in the discrimination of the nonhost plant.

In the La sensilla of *A. alcinous*, three cell types belonging to classes 1, 2, and 3 (Figure 5) responded to different compounds in the host and nonhost plant extracts. A class 3 cell responded to compounds in the nonhost plant extract, which differ from those in the host plant extract. Cells from classes 1 and 2 did not respond to the nonhost plant extracts and sucrose. Previously, we reported that in *A. alcinous*, aristolochic acid and sequoyitol evoke electrophysiological responses in two different cell types [18]. Hence, cells from classes 1 and 2 may respond to aristolochic acid and sequoyitol differentially. Comparison with other swallowtail butterflies revealed that La sensilla of *A. alcinous* appear similar to the female L1 sensilla of *P. xuthus* [19] based on the large number of sensilla and electrophysiological response to sugars. Both sensilla show no response to the disaccharide sucrose, and *P. xuthus* sensilla show no response to the monosaccharides fructose and glucose. Because sequoyitol is a monosaccharide derivative, cells from class 1 or class 2 may respond to sequoyitol in the La sensilla of *A. alcinous*.

*A. alcinous* larvae have three types of contact chemosensilla, one pair per mouthpart, namely LS, MS, and EP sensilla (Table 2). The three sensilla are considered to contain a few cells responding to the host plant extract of *A. debilis*. Substantial responses to 1 mM aristolochic acid were not observed in all sensilla. However, the LS sensillum contained one cell that responded with a phasic–tonic spike pattern of a single amplitude. Similarly, the LS sensillum contained one cell that responded to 15 mM sequoyitol. We could not determine whether the two cells responding to the host plant extract also responded to aristolochic acid or sequoyitol. Thus, whether the LS sensillum can recognize substances specific to the host plant remains unclear. In the maxilla, sucrose was detected by a cell in the MS sensillum but not in the LS sensillum. In the epipharynx, the EP sensillum contained one cell that strongly responded to sucrose and another that strongly responded to the nonhost plant extract (Figure 7). Response characteristics of the cell that strongly responds to the nonhost plant extract are categorized as the response patterns commonly found in a deterrent cell, i.e., slower increase in spike frequency and increased spike amplitude with time after latency [20,23,24]. The main difference was the contrast with the phasic–tonic spike pattern of the sugar response, as observed in response to 100 mM sucrose from the same sensilla. A deterrent cell can detect various compounds with an antifeedant effect on the insect [25]. For host plant recognition, LS, MS, and EP sensilla of *A. alcinous* larvae must function in concert by detecting feeding stimulants or feeding deterrents.

When sensilla between adults and larvae were compared, electrophysiological characteristics of La sensilla and LS sensilla appeared to be similar in nature (Table 1 and Table 2). Responses to host plant extract and sucrose stimulation of Lb and L sensilla appeared similar to those of MS sensilla (Table 1 and Table 2). Our findings suggest that chemosensory receptors with similar ligands, including host and nonhost plant compounds, are present in adults as well as larvae.

Although adults and larvae have different types of sensilla, the roles of the sensilla are probably associated with host selection. Our findings regarding the distribution and electrophysiological responses of tarsal sensilla in adults indicate that the La sensilla are specialized in oviposition during host selection. Female sensilla show a much greater number of La than that of Lb and S. A comparison between adult and larva sensilla revealed that La sensilla and LS sensilla show similar responses to the host plant compounds aristolochic acid and sequoyitol. Although larvae have only one pair of LS sensilla, they can detect host plant compounds during host selection. Sensilla other than La and LS, including female Lb and S, male L and S, and larval MS and EP respond to both host plant compounds and sucrose. These sensilla are considered important for detecting sugars during oviposition and feeding. Notably, EP sensilla strongly responded to nonhost plant extracts, suggesting that they exclude nonhost plants during diet selection.

Phytophagous insects regulate feeding by balancing the feeding stimulants and deterrents detected by taste cells [25]. Sucrose is a common feeding stimulant. However, a combination of aristolochic acid and sequoyitol may be a specific feeding stimulant for *A. alcinous* larvae. Additionally, the detection of bitter substances in nonhost plants is an important factor in food recognition. The larval process of host plant detection appears to be similar to oviposition behavior in adults. Although aristolochic acid is a major oviposition stimulant in *A. alcinous*, it is a deterrent for other lepidopteran species [26,27]. These findings suggest that adults and larvae of *A. alcinous* have evolved specialized chemosensory systems for detecting host plant compounds as stimulants for oviposition and feeding.

## Figures and Tables

**Table 1:** Number of cells responding to various stimulants in *Atrophaneura* *alcinous* adults.

Type of Sensilla	0.1 gle/mL *Aristolochia debilis*	0.1 gle/mL *Citrus* spp.	100 mM Sucrose	200 mM NaCl
Female La (70)	2	1	0	1
Female Lb (3–5)	2	1	2	1
Female S (6–8)	2	1	1	1
Male L (6–8)	2	1	2	1
Male S (6–8)	2	1	1	1

La: long-type sensilla a; Lb: long-type sensilla b; S: short-type sensilla; L: long-type sensilla. Numbers in parentheses indicate the total number of sensilla in the fifth tarsomere of the foreleg. At least 16 sensilla from 8 males and 32 sensilla from 16 females were electrophysiologically recorded. Methanolic extracts of host plant *Aristolochia debilis* and nonhost plant *Citrus* spp. “gle/mL” indicates gram leaf equivalent in 1 mL of control solution.

**Table 2:** Number of cells responding to various stimulants in *Atrophaneura alcinous* larvae.

Type of Sensilla	0.1 gle/mL *Aristolochia debilis*	0.1 gle/mL *Citrus* spp.	100 mM Sucrose	15 mM Sequoyitol	1 mM Aristolochic Acid
LS (1)	2	1	0	1	1
MS (1)	2	1	1	0	1
EP (1)	2	1	1	1	1

LS: lateral styloconic sensilla; MS: medial styloconic sensilla; EP: epipharyngeal sensilla. Numbers in parentheses indicate the total pairs of sensilla per individual mouthpart. At least 32 sensilla from 16 individuals were electrophysiologically recorded. Methanolic extracts of host plant *Aristolochia debilis* and nonhost plant *Citrus* spp. “gle/mL” indicated gram leaf equivalent in 1 mL of control solution.

## Data Availability

Data can be provided on request from the corresponding author.
